# Dextrose with Insulin During Neonatal Resuscitation for Prolonged Asphyxia in a Near-Term Ovine Model: A Proof-of-Concept Study

**DOI:** 10.3390/children13010050

**Published:** 2025-12-30

**Authors:** Praveen Chandrasekharan, Arun Prasath, Sylvia Gugino, Justin Helman, Lori Nielsen, Nicole Bradley, Mausma Bawa, Clariss Blanco, Mary Divya Kasu, Hamza Abbasi, Munmun Rawat, Jesse Slone

**Affiliations:** 1 Division of Neonatology, Department of Pediatrics, University at Buffalo, 1001 Main Street, Buffalo, NY 14068, USA; sfgugino@buffalo.edu (S.G.); jhelman@buffalo.edu (J.H.); lnielsen@buffalo.edu (L.N.); nkbradle@buffalo.edu (N.B.); mausmaba@buffalo.edu (M.B.); hamzaabb@buffalo.edu (H.A.); munmunra@buffalo.edu (M.R.); jslone@buffalo.edu (J.S.); 2 Division of Neonatology, Department of Pediatrics, UT Southwestern, Dallas, TX 75390, USA; arun.prasath@utsouthwestern.edu; 3 Division of Neonatology, Department of Pediatrics, Harlem Hospital, New York, NY 10037, USA; blancoc1@nychhc.org; 4 Division of Neonatology, Department of Pediatrics, University of Maryland, College Park, MD 20742, USA; mkasu@som.umaryland.edu; 5 Division of Genetics, Department of Pediatrics, University at Buffalo, 1001 Main Street, Buffalo, NY 14068, USA

**Keywords:** prolonged asphyxia, perinatal asphyxia, dextrose-insulin, metabolic resuscitation, ovine model, neonatal resuscitation, cardiac arrest

## Abstract

**Highlights:**

**What is the main findings?**

- In this proof-of-concept study using a near-term ovine model of prolonged asphyxia cardiac arrest, co-administration of dextrose and insulin with epinephrine was associated with a higher incidence of return of spontaneous circulation (ROSC) compared to epinephrine alone.

**What is the implication of the main findings?**

- Administration of dextrose/insulin with epinephrine may offer therapeutic benefits for cardiac resuscitation following prolonged perinatal asphyxia. However, due to the small sample size and lack of statistical significance, larger translational studies are necessary to confirm these findings and establish the clinical potential of this metabolic resuscitation strategy.

**Abstract:**

**Background**: Neonatal myocytes rely predominantly on glycolytic metabolism for survival during hypoxic conditions. During asphyxia, metabolic pathway dysregulation impairs cardiac myocyte contractility. Co-administration of dextrose and insulin may help restore metabolic homeostasis and improve cardiac function. **Methods**: Following blinded randomization and instrumentation, near-term lambs (138–140 days gestational age) were asphyxiated by umbilical cord occlusion until complete cardiac arrest, followed by 7 min of continued arrest to model severe asphyxia. Return of spontaneous circulation (ROSC) was defined as heart rate ≥ 100 beats per minute (bpm) and diastolic blood pressure ≥ 20 mmHg. **Results**: The incidence of ROSC was 3/6 in the control group compared to 5/5 in the experimental group receiving dextrose–insulin therapy, although this difference did not reach statistical significance. **Conclusions**: In this proof-of-concept study using a near-term ovine model of prolonged asphyxial cardiac arrest, dextrose and insulin co-administered with epinephrine were associated with improved ROSC rates although could be an association. Larger studies are needed to confirm these findings and evaluate clinical translation

## 1. Introduction

The transition from fetal to neonatal glucose homeostasis is a dynamic and often unpredictable process [1]. In utero, the fetus maintains lower blood glucose levels and operates at a lower metabolic rate than in postnatal life. Fetal glucose regulation is tightly controlled by placental glucose transfer, which supports critical brain development and promotes hepatic glycogen storage [1]. Following birth, placental supplementation ceases abruptly, and transitional hypoglycemia occurs commonly during the early neonatal period. Guidelines established by Adamkin et al., on behalf of the American Academy of Pediatrics Committee on the Fetus and Newborn (AAP-COFN), provide a framework for monitoring and managing neonatal hypoglycemia in high-risk clinical settings [1]. However, in newborns requiring resuscitation, particularly in the context of acidosis, hyperkalemia, and metabolic derangement, glucose metabolism and substrate utilization may follow substantially different pathways.

Neonatal cardiac myocytes are uniquely adapted to tolerate hypoxic conditions by relying on glycolytic metabolism [2,3,4]. During perinatal asphyxia, severe metabolic pathway dysregulation occurs, impairing myocardial contractility and energy production. Under anaerobic conditions, glucose consumption increases markedly as the myocardium attempts to maintain adenosine triphosphate (ATP) production via glycolysis. Despite these compensatory mechanisms, prolonged asphyxia leads to substrate depletion, acidosis, and progressive myocardial dysfunction, significantly reducing the likelihood of successful resuscitation.

Current neonatal resuscitation guidelines from the Neonatal Resuscitation Program (NRP) recommend continuing resuscitation efforts for at least 20 min or until return of spontaneous circulation (ROSC) is achieved [5]. However, outcomes for infants requiring prolonged resuscitation remain poor. A systematic review by Foglia et al. demonstrated that newborns still requiring resuscitation at 10 min after birth face a substantially elevated risk of mortality and neurodevelopmental impairment (NDI), though survival without moderate or severe NDI remains possible in a subset of cases [6]. Importantly, no single duration of resuscitation uniformly predicts survival or neurologically intact survival, underscoring the need for adjunctive metabolic therapies that may improve resuscitation success.

Co-administration of dextrose with insulin has been proposed as a metabolic resuscitation strategy to enhance myocardial glucose uptake, improve cardiac contractility, and restore cellular energy balance during resuscitation from asphyxial cardiac arrest. Insulin facilitates glucose transport into cardiomyocytes and may enhance glycolytic ATP production under anaerobic conditions. However, the efficacy and safety of this approach in the setting of neonatal asphyxial cardiac arrest have not been well studied.

The aim of this proof-of-concept study was to evaluate the effect of dextrose and insulin co-administration, in addition to standard epinephrine therapy, on the incidence and timing of ROSC, as well as on hemodynamic parameters and gas exchange, in a near-term ovine model of prolonged asphyxial cardiac arrest.

## 2. Materials and Methods

### 2.1. Ethics and Animal Model

This study was conducted in accordance with ARRIVE guidelines (supplemental attached) following approval by the Institutional Animal Care and Use Committee of the University at Buffalo (PED 10085N). We used near-term time-dated pregnant ewes (Polypay breed) at 138–140 days of gestation (term = 150 days). These lambs are equivalent to 36–37 weeks human gestation.

### 2.2. Surgical Preparation and Instrumentation

With the ewe under general anesthesia, the lambs were partially exteriorized and intubated with a cuffed 4.5 mm endotracheal tube (ETT). Fetal lung liquid was drained, and the ETT was occluded to prevent air entry and maintain fetal physiology. A jugular venous catheter was placed for intravenous access and blood sampling. The right carotid artery was catheterized for continuous arterial blood pressure monitoring and arterial blood gas sampling. Ultrasonic flow transducers (Transonic Systems Inc., Ithaca, NY, USA) were positioned around the left common carotid artery and main pulmonary artery to continuously monitor blood flow as indicators of systemic and pulmonary hemodynamics, respectively.

### 2.3. Induction of Asphyxia and Cardiac Arrest

Definition of prolonged asphyxia: Based on the systematic review by Foglia et al., which identified an Apgar score of zero at 10 min as a critical threshold for adverse outcomes [6], we sought to model clinically relevant prolonged perinatal asphyxia.

Asphyxia was induced by umbilical cord occlusion and maintained until complete cardiac arrest occurred, defined as: (1) heart rate of zero, (2) absence of left carotid and pulmonary artery blood flow, and (3) isoelectric electrocardiogram (ECG). Following cardiac arrest, an additional 7 min of arrest were observed before initiating resuscitation to simulate severe, prolonged asphyxia.

### 2.4. Randomization and Blinding

Following complete cardiac arrest, lambs were randomized using a sealed-envelope method with blinded allocation to either the control or the experimental group. Investigators performing resuscitation were blinded to group assignment through the use of equivalent-volume sham treatment in the control group.

### 2.5. Resuscitation Protocol

**Figure 1:**
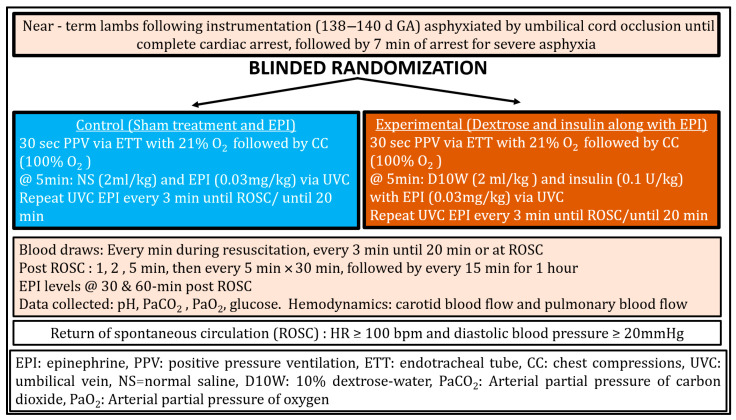
Design, Methodology and Resuscitation Protocol.

The resuscitation protocol is illustrated in Figure 1. In both groups, resuscitation was initiated with positive-pressure ventilation (PPV) via ETT using a T-piece resuscitator (Neo-Tee infant, Mercury Medical, Clearwater, FL, USA) with 21% oxygen at an initial pressure of 35/5 cm H_2_O. After 30 s of PPV, inspired oxygen was increased to 100%, and chest compressions (CC) were initiated at a 3:1 compression-to-ventilation ratio. Ventilation pressures were titrated to deliver target tidal volumes of 8–9 mL/kg and were monitored using a Philips NM3 respiratory monitor as previously described [7].

Control group (Sham and Epinephrine): At 5 min of resuscitation, lambs received sham treatment (normal saline 2 mL/kg) and epinephrine (0.03 mg/kg followed by a 3 mL normal saline flush) via umbilical venous catheter (UVC). Epinephrine alone (0.03 mg/kg) was repeated every 3 min until ROSC or for up to 20 min.

Experimental group (Dextrose and Insulin with Epinephrine): At 5 min of resuscitation, lambs received dextrose 10% (2 mL/kg) and insulin (0.1 U/kg of porcine insulin, Vetsulin, Merck, Rahway, NJ, USA) along with epinephrine (0.03 mg/kg followed by a 3 mL normal saline flush) via UVC. Epinephrine alone (0.03 mg/kg) was repeated every 3 min until ROSC or for up to 20 min. Porcine insulin is routinely used in preclinical ovine models and was administered intravenously.

### 2.6. Outcome Measures

Primary outcome: Our primary outcome was incidence and time to Return of Spontaneous Circulation (ROSC). ROSC was defined as sustained heart rate ≥ 100 beats per minute (bpm) and diastolic blood pressure ≥ 20 mmHg. The time to ROSC was recorded from the initiation of resuscitation.

Secondary outcomes: Left carotid artery blood flow served as a surrogate marker of myocardial contractility, reflecting approximately 75% of cardiac output in the ovine model. Pulmonary artery blood flow and systemic blood pressures were continuously monitored to characterize pulmonary and systemic hemodynamics throughout the observation period using AcqKnowledge Acquisition and Analysis Software (BIOPAC Systems, Goleta, CA, USA).

### 2.7. Blood Gas Analysis

Arterial blood gases were obtained at baseline, every minute during resuscitation until ROSC, and post-ROSC at 1, 2, and 5 min, followed by every 5 min for the first 30 min, and subsequently every 15 min for an additional hour to assess gas exchange and metabolic parameters [8].

### 2.8. Statistical Analysis and Sample Size Estimation

As no prior studies have evaluated metabolic adjuncts during resuscitation from prolonged asphyxia in a neonatal large animal model, sample size estimation was based on preliminary data and established methodologies for pilot animal studies. To detect an average time difference of 1 min to achieve ROSC with a standard deviation of 0.5 min, α = 0.05, and power = 0.80, a minimum of five lambs per group was required. Using a Markov Chain Monte Carlo (MCMC) approach for pilot animal experiments, five to six animals per group provides statistical power ≥80% even for small effect sizes [9]. This represents an estimate for a proof-of-concept study to inform future adequately powered investigations.

Following completion of the study a post hoc power analysis was performed using time to ROSC as the primary outcome. Based on the observed difference in mean time to ROSC (control: 800 s vs. experimental: 424 s, difference = 376 s, SD = 54 s) with 11 subjects distributed in a 6:5 ratio, the achieved power was ≥0.80 with α < 0.05 using Vanderbilt Power Size Calculation software (version 3.1.6, Nashville, TN, USA). These could help for future prospective randomized controlled trials.

The Kolmogorov–Smirnov test was used to assess normality of data distribution. Demographic and outcome data are presented as frequencies and percentages for categorical variables, and as mean ± standard deviation or median (interquartile range) for continuous variables based on distribution. Chi-square test, unpaired *t*-test, ANOVA, and non-parametric tests (Mann–Whitney U, Kruskal–Wallis) were applied as appropriate. Statistical analyses were performed using SPSS (version 31, IBM, Armonk, NY, USA) and SAS (version 9.4, SAS Institute, Cary, NC, USA). A *p*-value < 0.05 was considered statistically significant.

## 3. Results

### 3.1. Baseline Characteristics

We used 11 near-term lambs randomized to either the control group (N = 6) or experimental group (N = 5), as shown in Table 1. There were no significant differences in baseline characteristics between groups. Following umbilical cord occlusion, all lambs progressed to complete cardiac arrest (absence of blood flow and heart rate). After the onset of complete cardiac arrest, an additional 7 min of arrest were observed before initiating resuscitation, as described in the Methods. The total time from cord occlusion to complete cardiac arrest was 26 ± 4 min in the control group and 24 ± 7 min in the experimental group (*p* = 0.565).

### 3.2. Incidence and Time to Return of Spontaneous Circulation

The primary outcome data are summarized in Table 2. All five lambs (5/5) in the experimental group (dextrose/insulin and epinephrine) achieved ROSC, compared to three of six lambs (3/6) in the control group (epinephrine alone). Although this difference did not reach statistical significance (*p* = 0.18), it represents a clinically meaningful trend toward improved resuscitation success with metabolic support. Comparing the ROSC times in controls, 400 ± 53 s (3/6) vs. 424 ± 54 s (5/5), *p* = 0.57 with effect size of d 0.45, there was no statistically significant difference between the two groups, though the moderate effect size suggests a potentially meaningful clinical difference that may be underpowered to detect.

When the time to ROSC was analyzed as a continuous variable, including all 11 lambs (those achieving ROSC and those not achieving ROSC within 20 min) (Table 2). Lambs that did not achieve ROSC were assigned a time of 1200 s (20 min). The median time in the control group was 475 (IQR 375, 1200) seconds, compared with 374 (IQR 372, 394) seconds.

### 3.3. Blood Glucose Levels

**Figure 2:**
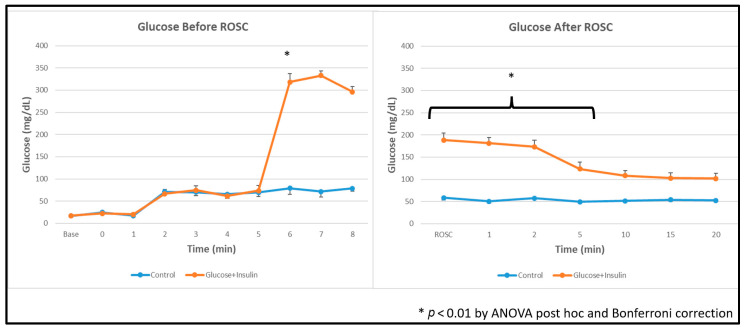
Blood glucose concentrations during and after resuscitation. Data are presented as mean ± standard deviation. Blood glucose levels were higher in dextrose and insulin group after administration and after ROSC. * *p* < 0.01 by ANOVA with Bonferroni post hoc correction comparing experimental vs. control groups.

Blood glucose concentrations before and after resuscitation are shown in Figure 2. Despite insulin co-administration, post-ROSC blood glucose levels remained significantly elevated in the experimental group compared to the control group (*p* < 0.01 by ANOVA with Bonferroni correction). 

### 3.4. Hemodynamic Parameters

**Figure 3:**
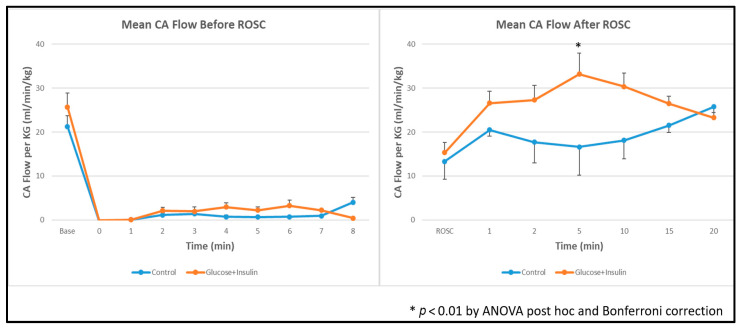
Left carotid artery blood flow during and after resuscitation. Data are presented as mean ± standard deviation. There was higher carotid artery blood flow in the dextrose and insulin group post ROSC at 5 min. * *p* < 0.01 at 5 minutes post-ROSC by ANOVA with Bonferroni post hoc correction comparing experimental vs. control groups. ROSC: Return of spontaneous circulation (ROSC).

Carotid Blood Flow: Post hoc ANOVA analysis showed significantly higher left carotid artery blood flow in the experimental group at 5 min post-ROSC (Figure 3, *p* < 0.01), suggesting enhanced myocardial contractility and systemic perfusion following dextrose–insulin therapy. By 20 min post-ROSC, carotid blood flow had normalized and was comparable between groups.

**Figure 4:**
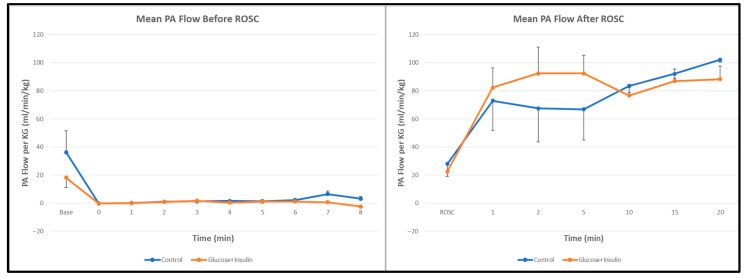
Pulmonary artery blood flow during and after resuscitation. No significant differences were observed between control and experimental groups. Data are presented as mean ± standard deviation.

Pulmonary Blood Flow: Pulmonary artery blood flow before and after ROSC was similar between groups (Figure 4, *p* = 0.67), suggesting that the hemodynamic effects of dextrose–insulin therapy were primarily systemic rather than pulmonary in distribution.

### 3.5. Blood Pressure

During resuscitation, systolic and diastolic blood pressures were 33 ± 5 mmHg and 15 ± 5 mmHg, respectively, in the control group, and 38 ± 6 mmHg and 15 ± 6 mmHg, respectively, in the experimental group. These differences were not statistically significant (*p* = 0.60).

### 3.6. Gas Exchange

**Figure 5:**
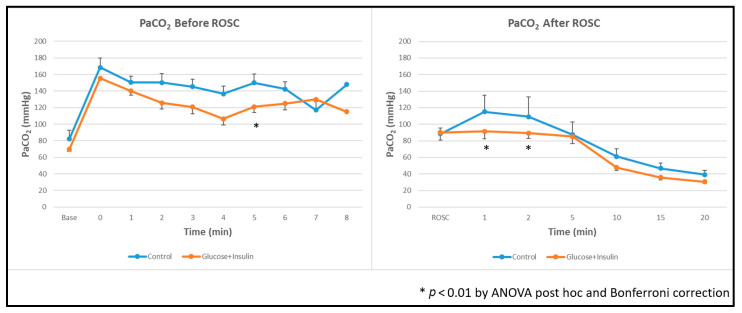
Arterial carbon dioxide levels during and after resuscitation. The experimental group demonstrated significantly lower PaCO_2_ levels throughout resuscitation and post ROSC. Data are presented as mean ± standard deviation. * *p* < 0.01 by ANOVA with Bonferroni post hoc correction comparing experimental vs. control groups. ROSC: Return of spontaneous circulation (ROSC).

**Ventilation:** The experimental group demonstrated significantly lower arterial carbon dioxide levels (PaCO_2_) both during and after resuscitation compared to the control group (Figure 5, *p* < 0.01).

**Figure 6:**
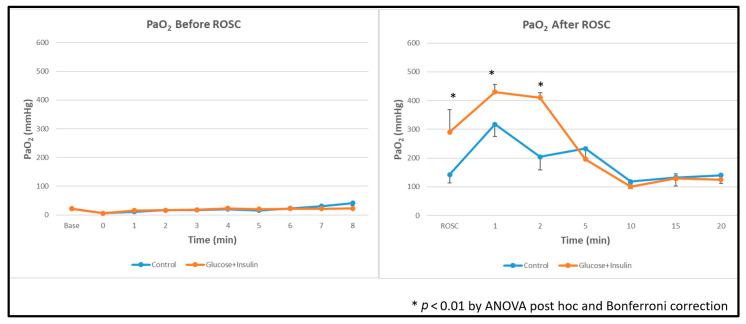
Arterial oxygen levels during and after resuscitation. The experimental group demonstrated significantly higher PaO_2_ levels post ROSC. Data are presented as mean ± standard deviation. * *p* < 0.01 by ANOVA with Bonferroni post hoc correction comparing experimental vs. control groups. ROSC: Return of spontaneous circulation (ROSC).

**Oxygenation:** Arterial oxygenation (PaO_2_) was significantly higher in the experimental group than in controls (Figure 6, *p* < 0.01), suggesting better overall tissue metabolism after ROSC.

## 4. Discussion

In this proof-of-concept study using a near-term ovine model of prolonged asphyxial cardiac arrest, co-administration of dextrose and insulin with epinephrine was associated with a higher incidence of ROSC (5/5 vs. 3/6) Although the difference in ROSC incidence did not reach statistical significance due to the small sample size, the experimental group demonstrated improved early post-ROSC hemodynamics, including higher carotid blood flow at 5 min post-ROSC and better gas exchange, with lower PaCO_2_ and higher PaO_2_. These findings suggest that metabolic support with dextrose–insulin may enhance myocardial recovery and cardiopulmonary function following severe perinatal asphyxia.

### 4.1. Glucose Metabolism and the Asphyxiated Myocardium

**Figure 7:**
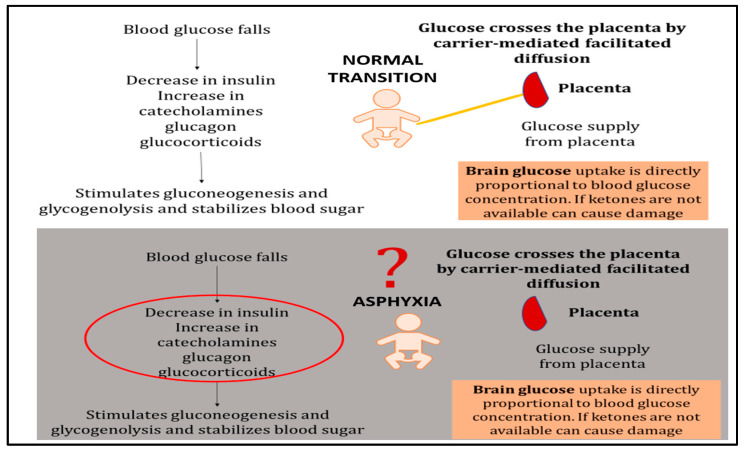
Glucose metabolism during normal neonatal transition versus asphyxia-induced dysregulation. *Normal transition*: Once the umbilical cord is cut, placental glucose supply ceases and blood glucose falls, triggering a decrease in insulin and compensatory increases in catecholamines, glucagon, and glucocorticoids. These hormonal changes mobilize hepatic glycogen stores to maintain blood glucose levels. Brain glucose uptake is directly proportional to blood glucose concentration, ensuring adequate substrate delivery. *Asphyxiated newborn*: During asphyxia, normal metabolic mechanisms become dysregulated. Excessive catecholamine release and insulin deficiency lead to depletion of cellular glucose stores despite potentially elevated circulating glucose levels. The lack of adequate insulin impairs substrate availability at the cellular level by preventing glucose transporter (GLUT) translocation to cell membranes. This creates a metabolic crisis in which glucose is circulating but cannot effectively enter cells to support energy production. Co-administration of dextrose with insulin may help restore cellular glucose availability and overcome this uptake barrier.

The fetal myocardium operates in a relatively hypoxic environment and relies predominantly on glycolytic metabolism for energy production [2,3,4]. During perinatal asphyxia, this metabolic vulnerability is exacerbated by substrate depletion, progressive acidosis, and impaired cellular energy balance. Blood glucose serves as the primary substrate for both the brain and heart during resuscitation (Figure 7).

**Figure 8:**
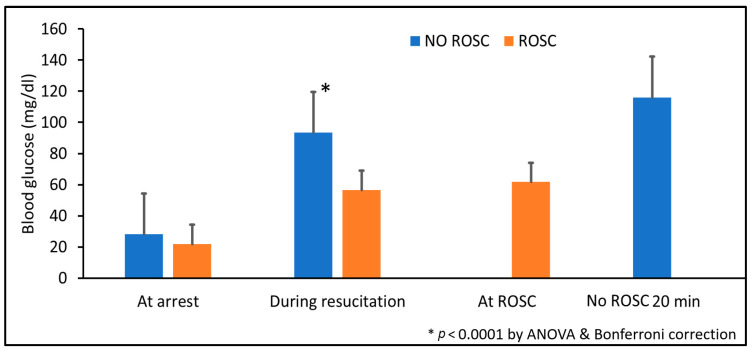
Blood glucose levels in lambs achieving versus not achieving ROSC in prior pilot studies. Blood glucose concentrations were measured at cardiac arrest, during resuscitation, at ROSC (for survivors), and at 20 minutes (for non-survivors). Data are presented as mean ± standard deviation. * *p* < 0.0001 by ANOVA with Bonferroni post hoc correction comparing ROSC vs. no ROSC groups. ROSC: Return of spontaneous circulation (ROSC).

Our previous pilot studies demonstrated that lambs failing to achieve ROSC had significantly higher blood glucose levels compared to those achieving ROSC (Figure 8), suggesting impaired cellular glucose uptake despite adequate circulating substrate, a phenomenon consistent with stress-induced insulin resistance and metabolic dysfunction during severe asphyxia.

The neonatal heart’s dependence on glucose becomes particularly critical during prolonged asphyxia when oxygen delivery is severely compromised. Under these conditions, glycolysis becomes the primary, if not the only means of adenosine triphosphate (ATP) production. However, asphyxia-induced metabolic derangements impair glucose transport into cardiomyocytes, creating an energy crisis that undermines myocardial contractility and reduces the likelihood of successful resuscitation [2,3,4].

### 4.2. Clinical Context: Duration of Resuscitation and the Need for Metabolic Adjuncts

Current Neonatal Resuscitation Program (NRP) guidelines recommend continuing resuscitation for at least 20 min or until ROSC is achieved [5]. A systematic review by Foglia et al. demonstrated that newborns with an Apgar score of 0 at 10 min (indicating absent heart rate) face substantially elevated risks of mortality and neurodevelopmental impairment, though survival without severe disability remains possible [6]. The variability in asphyxia duration and the unpredictable response to standard resuscitation underscore the critical need for adjunctive therapies that can enhance myocardial recovery and improve outcomes.

In our model, asphyxia was induced by umbilical cord occlusion until complete cardiac arrest, followed by an additional 7 min of arrest, resulting in approximately 25 min of total asphyxia time in both groups. This duration models the severe end of the clinical spectrum and represents cases where conventional resuscitation may be insufficient. The administration of dextrose and insulin at 5 min of resuscitation, combined with standard epinephrine therapy, resulted in ROSC at a median of 424 s in the experimental group.

### 4.3. Mechanistic Hypothesis: The RISK Pathway and Cardio Protection (Figure 9)

Speculative section without evidence from the study: We hypothesize that the observed improvements in myocardial recovery may involve activation of the Reperfusion Injury Salvage Kinase (RISK) pathway, a well-characterized pro-survival signaling cascade in adult cardiac ischemia–reperfusion injury [10,11,12]. The RISK pathway includes two principal arms: the phosphatidylinositol 3-kinase/protein kinase B (PI3K/Akt) pathway and the extracellular signal-regulated kinase 1/2 (ERK1/2) pathway.

**Figure 9:**
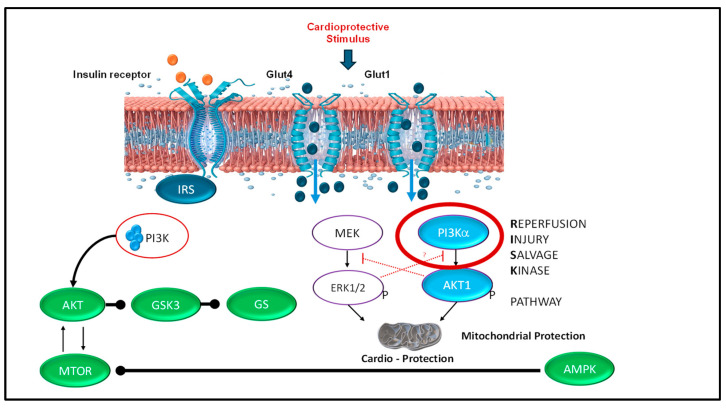
Reperfusion Injury Salvage Kinase (RISK) pathway: proposed mechanism of insulin-mediated cardioprotection. The RISK pathway is well-established in adult cardiac ischemia–reperfusion injury as a pro-survival signaling mechanism. We speculate this pathway may be activated by insulin administration during neonatal resuscitation, potentially protecting the heart and brain from reperfusion injury following prolonged asphyxia. *Mechanism:* Insulin binds to insulin receptors on the cardiomyocyte cell surface, initiating two parallel pro-survival signaling cascades: (1) PI3K/Akt pathway: Activation of phosphatidylinositol 3-kinase (PI3K) leads to phosphorylation and activation of protein kinase B (Akt). Activated Akt phosphorylates and inhibits glycogen synthase kinase-3 (GSK3), a key pro-apoptotic enzyme; when inhibited, GSK3 promotes cell survival. (2) ERK1/2 pathway: Activation of MEK leads to phosphorylation and activation of extracellular signal-regulated kinases 1 and 2 (ERK1/2), providing additional pro-survival signaling. Mitochondrial protection: Both pathways converge on mitochondria, where their combined action inhibits opening of the mitochondrial permeability transition pore (mPTP), a critical event in reperfusion-induced cell death. By preventing mPTP opening, these pathways preserve mitochondrial integrity and prevent cardiomyocyte death [12]. This mechanism remains speculative in the current study, as no molecular analyses were performed to confirm RISK pathway activation in our neonatal ovine model.

Insulin binds to insulin receptors on cardiomyocyte cell membranes, activating PI3K, which in turn phosphorylates and activates Akt. Activated Akt subsequently phosphorylates and inhibits glycogen synthase kinase-3β (GSK3β), a key mediator of cell death. Simultaneously, the ERK1/2 pathway is activated through MEK phosphorylation. Both pathways converge on the mitochondria, where they inhibit opening of the mitochondrial permeability transition pore (mPTP)—a critical event in reperfusion-induced cell death. By preventing mPTP opening, RISK pathway activation preserves mitochondrial integrity, maintains cellular energy production, and reduces cardiomyocyte apoptosis [13].

We speculate that dextrose and insulin administration during resuscitation may activate similar cardioprotective mechanisms in the neonatal heart. However, this hypothesis remains untested in the current study, as no molecular assays of PI3K/Akt signaling, GSK3β phosphorylation, or mPTP function were performed.

### 4.4. Clinical Context: Glucose–Insulin–Potassium (GIK) Therapy

The concept of metabolic support during myocardial ischemia is not new. Glucose–insulin–potassium (GIK) therapy has been investigated in adults with acute myocardial infarction for over 50 years, with proposed benefits including enhanced myocardial glucose uptake, improved contractility, anti-arrhythmic effects through membrane stabilization, and reduced free fatty acid metabolism [14]. In neonates, GIK therapy has been used as salvage therapy for severe hyperkalemia, particularly in the context of acidosis [15].

In our study, we omitted potassium supplementation due to concerns about hyperkalemia in the immediate post-arrest period when renal function is compromised and urine output is absent. Instead, we focused on dextrose (2 mL/kg of 10% solution) and porcine insulin (0.1 U/kg) to facilitate glucose uptake and provide metabolic substrate to the energy-depleted myocardium. Our findings suggest that even without potassium, dextrose–insulin therapy may confer significant hemodynamic benefits during neonatal resuscitation.

It is important to note that the pathophysiology differs substantially between adult myocardial infarction (acute coronary occlusion with regional ischemia) and neonatal asphyxia (global hypoxemia and ischemia affecting multiple organ systems). Nevertheless, the fundamental metabolic crisis, substrate depletion, and impaired energy production are shared, suggesting that metabolic support strategies may be translatable to neonates.

### 4.5. Hemodynamic and Gas Exchange Findings

The experimental group demonstrated significantly higher left carotid artery blood flow at 5 min post-ROSC (Figure 3), with normalization by 20 min. In the ovine model, carotid blood flow reflects approximately 75% of cardiac output and serves as a reliable surrogate for myocardial contractility. This early hemodynamic advantage suggests that dextrose–insulin therapy may enhance myocardial function during the critical early post-resuscitation period, potentially improving end-organ perfusion and reducing the risk of secondary injury.

Interestingly, pulmonary artery blood flow was comparable between groups (Figure 4), suggesting that the hemodynamic effects of dextrose–insulin therapy were predominantly systemic rather than pulmonary.

### 4.6. Paradoxical Hyperglycemia

Despite insulin co-administration, post-ROSC blood glucose levels remained significantly elevated in the experimental group (Figure 2). This paradoxical hyperglycemia likely reflects the complex metabolic milieu of resuscitation, including: (1) exogenous dextrose administration (2 mL/kg of 10% solution glucose bolus); (2) endogenous catecholamine release during asphyxia and resuscitation, which stimulates hepatic glycogenolysis and gluconeogenesis while inducing insulin resistance; (3) delayed cellular glucose uptake due to asphyxia-induced metabolic suppression and impaired insulin signaling; and (4) the pharmacodynamics of intravenous porcine insulin in the setting of critical illness.

The clinical significance of transient hyperglycemia in this context remains unclear. While hyperglycemia has been associated with poor neurological outcomes in neonatal hypoxic–ischemic encephalopathy [16], it is uncertain whether the hyperglycemia is causative or simply a marker of severity of illness. In our model, animals receiving dextrose–insulin had better outcomes despite higher glucose levels, suggesting that the metabolic benefits—enhanced myocardial glucose uptake and energy production—may outweigh potential glycemic concerns in the acute resuscitation phase. Nonetheless, post-resuscitation glucose management and its impact on neurological outcomes warrant careful investigation in future studies.

### 4.7. Timing and Pharmacokinetics of Insulin

We administered porcine insulin (Vetsulin) intravenously at a dose of 0.1 U/kg at 5 min of resuscitation. Porcine insulin has been used extensively in preclinical ovine models and has pharmacokinetic properties similar to human insulin. The intravenous route was chosen to ensure rapid onset of action, as subcutaneous absorption would be unreliable during low-flow states associated with chest compressions.

The timing of administration—at 5 min, coinciding with the first epinephrine dose—was strategic. By this point, chest compressions had been ongoing for approximately 4.5 min, potentially priming the circulation and facilitating drug delivery to target tissues. The mean time to ROSC in the experimental group was approximately 7 min after initiation of resuscitation, suggesting that the metabolic effects of dextrose–insulin manifested within minutes of administration.

It is important to note that during chest compressions, cardiac output is only 25–33% of normal, and blood flow is preferentially directed to the brain, heart, and adrenal glands. This selective perfusion may actually enhance drug delivery to the myocardium while reducing systemic distribution, potentially explaining the rapid onset of effect. The concurrent surge of endogenous epinephrine and norepinephrine from the adrenal glands, combined with exogenous epinephrine administration, creates a complex neuroendocrine milieu that likely influences insulin action and glucose metabolism.

### 4.8. Limitations

This proof-of-concept study has several important limitations. The small sample size (N = 11) limits statistical power, and the observed difference in ROSC incidence 5/5 vs. 3/6 while clinically meaningful, did not reach statistical significance (*p* = 0.18). We did not include a dextrose-alone control group, preventing us from isolating insulin’s specific contribution to the observed benefits. No molecular or tissue-level analyses were performed to confirm the proposed RISK pathway mechanism, making Figure 9 entirely speculative. Species differences between ovine and human neonates, along with variations in glucose homeostasis and insulin sensitivity, must be considered when extrapolating these findings. We did not assess markers of reperfusion injury (biochemical markers, cardiac imaging, or histopathology), and the short observation period (1 h post-ROSC) precluded evaluation of long-term survival, neurological outcomes, or potential adverse effects such as rebound hypoglycemia. The paradoxical post-ROSC hyperglycemia observed in the experimental group raises concerns, given the known association between hyperglycemia and neurological injury in neonatal hypoxic–ischemic encephalopathy, though whether this represents a causal relationship or marker of illness severity remains unclear, although blood sugar levels seem to normalize within 1 h of intervention.

### 4.9. Future Directions

Larger, adequately powered studies are planned with additional treatment arms (dextrose-alone and insulin-alone) to isolate individual drug contributions. Dose–response studies will optimize dextrose and insulin dosing, ratios, and timing to identify the most effective therapeutic window. Future investigations incorporating early electroencephalography, myocardial tissue analysis, immunohistochemistry for pathway components, and functional mitochondrial assays are planned to confirm whether RISK pathway activation validates the proposed mechanism. After successful testing with a larger sample size in animal models, well-designed clinical trials will be needed to assess safety, pharmacokinetics, and efficacy in human neonates.

## 5. Conclusions

In a near-term ovine model of prolonged asphyxial cardiac arrest, dextrose–insulin co-administration with epinephrine was associated with improved ROSC rates (5/5% vs. 3/6%, *p* = 0.18), with similar time to ROSC, enhanced early post-resuscitation hemodynamics (higher carotid blood flow at 5 min), and better gas exchange compared to epinephrine alone. These proof-of-concept findings suggest metabolic support may enhance cardiac resuscitation following severe perinatal asphyxia. However, the small sample size, lack of statistical significance in ROSC incidence, absence of mechanistic validation, and short observation period necessitates larger translational studies with comprehensive molecular analyses and long-term outcome assessments before clinical translation. If confirmed, dextrose–insulin therapy could represent a readily available, low-cost adjunct to improve outcomes in neonatal resuscitation.

## Figures and Tables

**Table 1:** Baseline Characteristics of the Lambs. Data is presented as mean and standard deviation or as N as specified.

Parameters	Control (N = 6) (Sham Treatment with Epinephrine)	Experimental (N = 5) (Dextrose + Insulin with Epinephrine)
Gestational age (days)	140 ± 2.0	140 ± 1.5
Birth weight (kg)	4.20 ± 1.2	4.23 ± 1.5
Sex (N)	M-3, F-3	M-2, F-3
Blood glucose at the start of resuscitation (mg/dL)	25.0 ± 4.7	22.5 ± 8.2

**Table 2:** Incidence and time to ROSC. ROSC: Return of spontaneous circulation (ROSC) was defined as heart rate ≥ 100 bpm and diastolic blood pressure ≥ 20 mmHg. As per ILCOR/AAP NRP recommendations, 20 min of resuscitation was performed if no ROSC was achieved. These times were included for power and sample size calculation. Data represented as mean and standard deviation. NA—Not appilcable.

Parameters	Control (N = 6) (Sham Treatment with Epinephrine)	Experimental (N = 5) (Dextrose + Insulin with Epinephrine)
ROSC	3/6 (50%)	5/5 (100%)
Time to ROSC (seconds)	400 ± 53	424 ± 54
Time: no ROSC (seconds)	1200 ± 0	NA

## Data Availability

Ongoing study. Data will be available upon reasonable request once the study is completed.

## Abbreviations

The following abbreviations are used in this manuscript:

DI	Dextrose and insulin
BG	blood glucose
ROSC	Return of Spontaneous Circulation
RISK	Reperfusion Injury Salvage Kinase
IRS	Insulin Receptor Substrate
PI3K	Phosphatidylinositol 3-kinase
AKT	Protein Kinase B (also known as PKB)
GSK3	Glycogen Synthase Kinase-3
GS	Glycogen Synthase
MEK	Mitogen-activated protein kinase kinase (also known as MAP2K)
ERK1/2	Extracellular signal-regulated kinases 1 and 2
mTOR	Mechanistic Target of Rapamycin
AMPK	AMP-activated protein kinase
Glut1	Glucose transporter type 1
Glut4	Glucose transporter type 4
